# Historical Impact of Baron Dominique-Jean Larrey: The Father of Modern Military Surgery

**DOI:** 10.7759/cureus.108744

**Published:** 2026-05-12

**Authors:** Jatin S Dhamrait, Avrie D Barthel, Henry J Styron, Joshua Garrett, Rushil Gupta, Tracy Si, Titilayo Shobayo, Tyler Lackland, Joseph Platz

**Affiliations:** 1 Department of Surgery, Saint Louis University School of Medicine, St. Louis, USA; 2 Department of Surgery, Loyola University Chicago Stritch School of Medicine, Chicago, USA

**Keywords:** amputation, dominique-jean larrey, flying ambulance, military surgery, napoleonic wars, triage

## Abstract

Baron Larrey was an 18th-century revolutionary in the field of military surgery. Serving as Napoleon Bonaparte’s trusted Surgeon-in-Chief, he utilized his inquisitive nature, alongside his experience, to develop impactful changes in the field, including the flying ambulance, the triage system, improvements in amputation procedures, and a new therapeutic outlook on hypothermia. His contributions toward improving military surgery led to both reduced soldier mortality and the utilization of surgical principles that we still use in modern medicine. His efforts earned him the title of “The Father of Modern Military Surgery”.

## Introduction and background

Baron Dominique-Jean Larrey (1766-1842) was one of the earliest pioneers of modern military surgery, laying down the foundations for emergency medical services and ambulatory care as the Surgeon-in-Chief of the French Army. From his humble beginnings during the French Revolution to his battlefield experience under Napoleon Bonaparte, Larrey made significant contributions to the humanistic care of wounded soldiers [1]. His dedication not only elevated the morale of his fellow soldiers, thereby strengthening the war effort, but also led to pioneering surgical innovations that remain influential to this day, such as the concept of triage, the use of heli ambulances, and wound sanitation [2,3]. Larrey’s military experience further paved the way for surgical advances in pericardiocentesis and amputation guidelines, elevating these approaches to fundamental modern procedures [2]. Collectively, these contributions established Larrey as a central figure in the evolution of both military and trauma surgery. Beyond technical innovation, Larrey's work represented an early shift in organized battlefield medicine. Many of the principles developed by Larrey continue to underpin modern trauma systems, emergency medical services, and disaster response protocols. This review aims to critically examine both the historical and clinical contributions of Baron Dominique-Jean Larrey and evaluate the lasting impact of his efforts on the development of contemporary trauma surgery, triage systems, and emergency medical services [4].

## Review

Methods

A narrative historical review regarding the medical and surgical contributions of Baron Dominique-Jean Larrey was conducted. Literature searches were performed using PubMed, Google Scholar, StatPearls, and the National Library of Medicine's historical medical archives. Search terms including: "Dominique-Jean Larrey", "military surgery", "triage", "ambulance volante", "therapeutic hypothermia", "amputation", and "pericardiotomy" were utilized to gather substantial evidence for the full manuscript. 

The inclusion of information was limited to articles and historical texts relevant to Larrey's clinical innovations. Both historical primary sources and modern secondary literature were included to provide historical context and establish clinical relevance. 

Historical background

Dominique-Jean Larrey was born in Baudéau, Hautes-Pyrénées, France, in 1766 [5]. He was orphaned at a young age and taken into the care of a priest, Abbé de Grasset [1,6]. At the age of 13, under the influence of surgeons including his brother, Claude François Hilaire Larrey, and his uncle, Alexis Larrey, he moved to Toulouse, France, to begin training in surgery at the Hospital of Grave [1,5,6]. His career began to ascend quickly. At the young age of 15, he was given the title of “dresser” and assigned to clean and dress surgical wounds; after a few short years, he was appointed House Surgeon [6]. At the age of 21, he traveled to Paris and took the military service entrance examination. He successfully underwent a six-month voyage to Newfoundland on the French Navy frigate Vigilante. However, this voyage marked his only service in the Navy due to episodes of severe seasickness [5,6].

Larrey returned to Paris at the height of the French Revolution in 1789 [1]. There, he trained under the well-known physician, Dessault, who was the chief surgeon at the Hôtel-Dieu Hospital [1,5,6]. After completing his formal training, he continued to study and refine his surgical skills at the Hôtel-Dieu de l'Invalides [1]. By 1792, Larrey had returned to military service and served as Chirurgien aide-major in the Army of the Rhine. While serving in this military capacity, he was inspired to create change, as he saw disorganized care for wounded soldiers result in irreparable harm and fatalities [6].

Upon returning from this military campaign, Larrey became Professor of Surgery at the Val de Grace Military Hospital in Paris [6]. He met Napoleon Bonaparte in 1794 and quickly gained the general's favor, traveling with him on campaigns to the Middle East, Germany, Poland, and Russia [6,7]. He achieved considerable success in this role and was named Surgeon-in-Chief of the French army in 1805 [7]. During his campaigns with Napoleon, he refined many medical advancements that would ultimately define his legacy. Much of Larrey’s work was influenced by his contemporary Jean-François Percy, another prominent military surgeon who contributed to the organization of battlefield medical transport and military surgical services [7]. While Percy emphasized the structured organization of the medical corps and transport, Larrey distinguished himself through the operational implementation of rapid evacuation systems and severity-based triage on the battlefield. Historians debate the influence of Percy and Larrey on the evolution of military medicine. Larrey's integration of rapid evacuation, triage, and equitable battlefield care significantly shaped the development of modern trauma systems. He is remembered for his profound impacts on both emergency medicine and modern military medicine [2,8].

Baron Dominique-Jean Larrey influenced the constantly evolving field of surgical medicine in several ways. On the battlefields of the French Revolution and Napoleonic Wars, Baron Larrey introduced the triage system for soldier care, emphasized urgent rather than delayed amputation, invented the first ambulance, and helped lay the foundation for therapeutic hypothermia and the management of pericardial effusion. These advancements can be credited for saving the lives of countless soldiers during the 18th and 19th centuries, while also laying the foundation for future surgical advancements and the expansion of theories still used in modern medicine [9].

Triage and battlefield prioritization

Larrey’s first impact on the care of soldiers was through his novel idea of a triage system, in which he prioritized care for the most severely wounded soldiers first [8,9]. The prioritization of injury severity over military rank led to operational principles that currently guide civilian emergency medicine, disaster response systems, and military combat casualty care [10,11]. This idea is believed to have come during his first experiences of war as a medical division chief around 1792 and emerged in stark contrast to the accepted practice of military medicine in which wounded soldiers were “left where they fell” and were gathered at the end of battle to receive treatment [9,11]. Not only did this previous method delay treatment by hours to days while also increasing infection risk, but soldiers’ need to self-evacuate or rely on untrained civilians for assistance in evacuation predisposed them to further injury. Moreover, high-ranking officials, regardless of the severity of their injuries, were treated first, followed by lower-ranking officials, even if their wounds were life-threatening. Larrey advocated dismantling this system, emphasizing that the most urgent wounds should be treated first, regardless of soldiers’ rank [7]. Thus, his ideas advanced humanism in wartime medicine and were applied in battles throughout his tenure as Surgeon-in-Chief. Importantly, Larrey's triage philosophy represented a departure from the medical practices of his era, which were based on social hierarchy.

Mobile battlefield medicine: the “ambulance volante”

**Figure 1:**
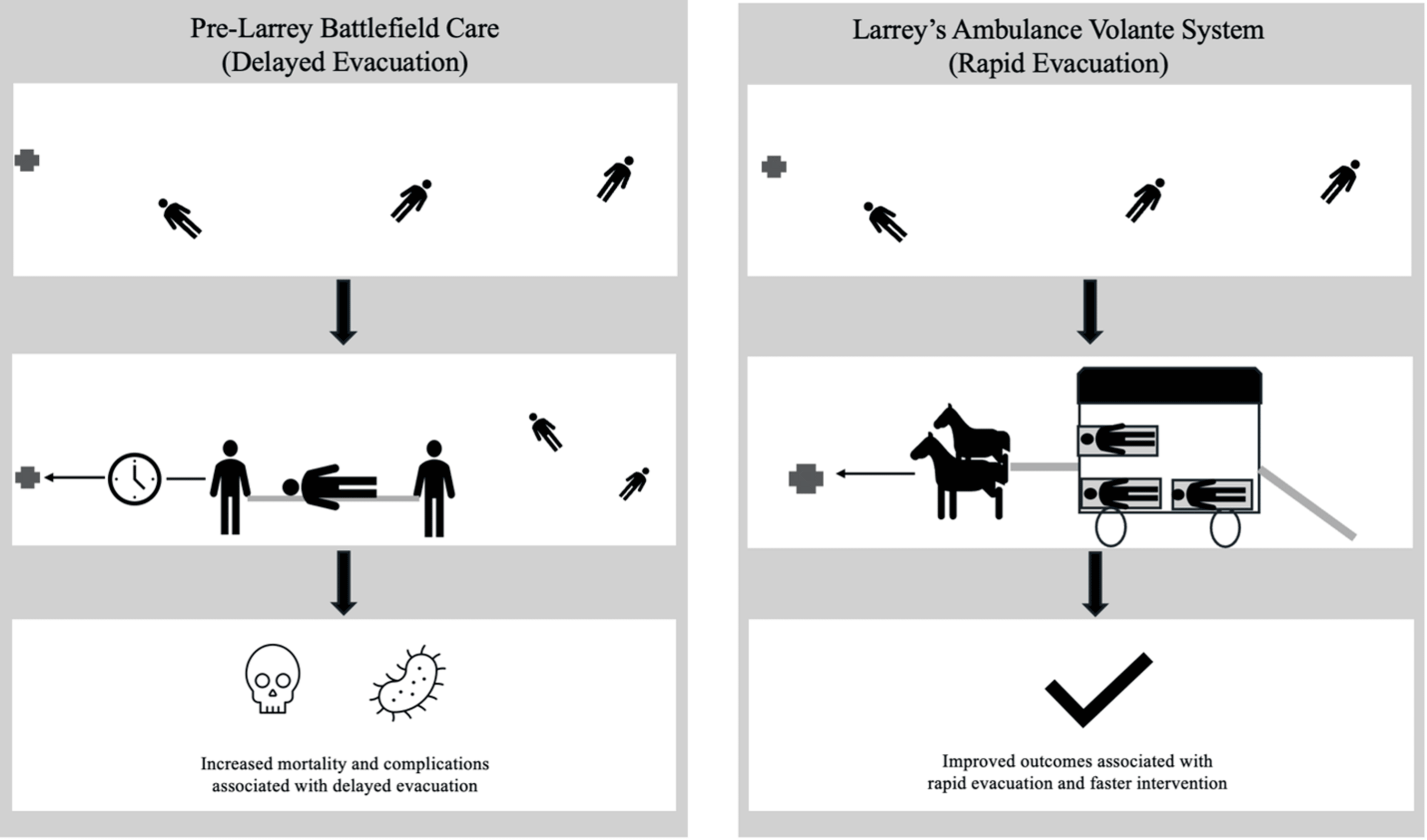
Comparison of battlefield evacuation before and after Larrey’s innovation Illustration demonstrating the evolution of battlefield evacuation systems. The figure highlights the contrast between delayed and manual transport. Arrows indicate the direction of transport, while the clock icon represents delays in care, which are associated with increased mortality and complications. Source: Created by the authors based on historical descriptions of Larrey’s system. Figure created using Microsoft PowerPoint (Microsoft Corporation, Redmond, WA).

Baron Larrey’s approach to mobile care, namely, the “ambulance volante” (flying ambulance), demonstrates advanced efficiency in injury management, as shown in Figure 1. This novel invention, introduced in 1809, removed injured soldiers from the front lines, pulling them out of the line of fire by horse-drawn wagons and placing them in a safe environment, as described in Larrey’s surgical memoirs [12]. These forms of transport were not just a means of moving patients but also carried crucial medical supplies to the battlefield, shortening the time before soldiers received time-sensitive, life-saving care. Arguably, the most shocking aspect of Larrey’s proposal was that the flying ambulances cared for soldiers on both sides of the battle, emulating justice and nonmaleficence. This not only highlights Larrey’s brilliant mind but also his dedication to caring for others before all else. Larrey’s invention revolutionized safety for those providing medical care, and it served as a model for trauma medicine for years to come [6]. The concept of rapid evacuation from the point of injury to definitive care mirrors the structure of modern emergency medical services [13]. This includes ground ambulance systems and aeromedical evacuation. Larrey's model demonstrated one of the earliest organized approaches to reducing delays in trauma care delivery.

Advancements in amputation techniques

**Figure 2:**
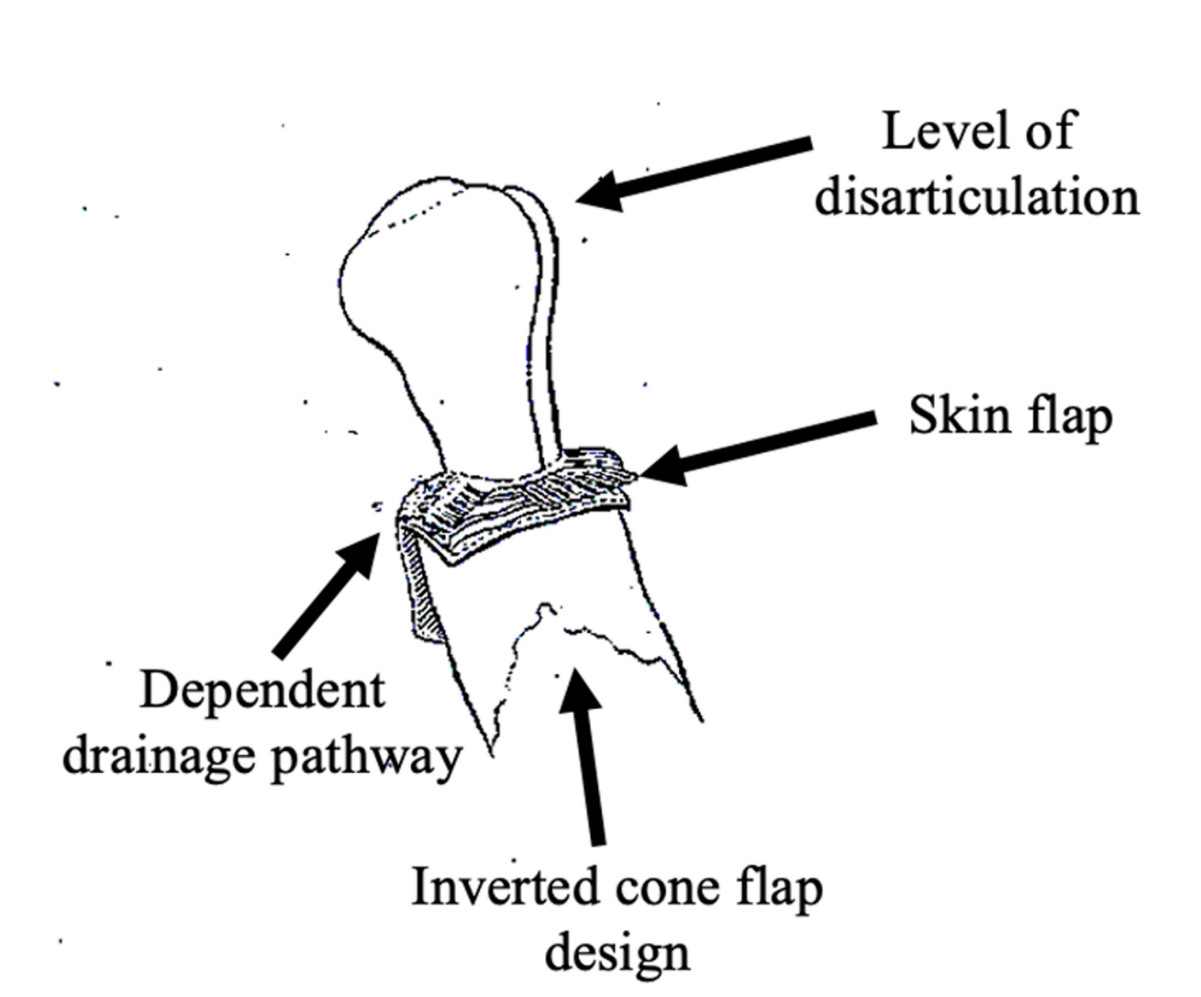
Shoulder disarticulation as described by Larrey. An annotated depiction of Baron Dominique-Jean Larrey’s surgical approach to shoulder disarticulation, highlighting the level of disarticulation, soft tissue flap design, and dependent drainage pathway to facilitate wound closure and reduce fluid accumulation. Source: Reference [12] (public domain).

Beginning in the late 1700s, Larrey introduced triage and ambulances and changed how amputations were managed after soldiers were wounded in battle. At the time, many wartime physicians believed that amputation should be delayed at least one to two days after the wound’s onset, allowing for “dead tissue” to be established before the procedure [14,15]. However, Larrey noticed higher infection and mortality rates in those receiving delayed amputations, leading to a push for earlier intervention. He believed that performing amputations within one day of injury would ultimately allow for faster wound healing by avoiding infection and inflammation, while also preventing prolonged suffering for soldiers and improving hemorrhage control [9,15]. This idea was applied mainly to wounds with injuries to major vessels, severe tissue damage, or major bone fractures, and was exemplified in the Battle of Borodino of 1812, in which he is reported to have performed approximately 200 amputations over the course of a single day [7,15]. Larrey advocated for early amputation following battlefield injuries rather than delayed, reporting lower mortality rates with this method [2,15]. Larrey's emphasis on rapid intervention parallels many principles of modern damage-control surgery [16]. Amongst this concept, early operative management is used to reduce hemorrhage, limit physiologic deterioration, and improve survival following severe traumatic injury. In addition, Larrey’s innovative approach to amputations included a newly proposed technique he called the “inverted cone,” as shown in Figure 2 [12]. This method allowed skin flaps at the end of the amputation site to be sewn together and subsequently dressed in gauze, thereby facilitating fluid drainage from the site [7]. Beyond amputation management, Larrey's surgical innovations extended into more complex operative interventions. He also contributed to early procedures related to the pericardium and thoracic cavity.

Combined with this new approach to amputation was Larrey’s belief in the need for wound washing and debridement to reduce the risk of postoperative infection. In this manner, he attempted to rid wounds of infection and unsalvageable tissue, while also utilizing more aqueous-based solutions for wound washing and avoiding the traditionally used ointments and salves for wound dressings [2,6].

Early contributions to pericardial surgery

One notable surgical advancement that began on the battlefield but found its way into civilian medicine was the management of pericardial effusion. Because true Cardiac Surgery during the early 19th century was essentially nonexistent, new surgical proposals involving intervention on the cardiovascular system were considered profound and extreme. Records indicate that Baron Larrey may have performed the first pericardiotomy on March 18, 1810; there is some debate as to whether he or Francisco Romero, a Barcelonian surgeon during Larrey’s time, performed the procedure first, but Larrey’s effort was the first to be accepted by the medical community [17]. His completion of a pericardiotomy for a patient with purulent pericarditis status post penetrating cardiac injury was revolutionary for his time, given the level of invasiveness for an organ that was not widely operated on. On February 22, 1824, he attempted treatment of another pericardial effusion, deviating from the established protocols involving ointment-containing gauze compression dressings [12]. Instead, Larrey removed the bandages and drained the pericardial fluid, a procedure named pericardiotomy. This time, the procedure was successful, ultimately leading to the patient's recovery and revealing to Larrey that the increased fluid in the pericardial sac directly correlated with the patient's discomfort. Larrey published his method for pericardiotomy in 1829, thereby sparking major advances in the treatment of pericardial effusions over the next century [12].

Observations on therapeutic hypothermia

Larrey’s final notable contribution to wartime surgical medicine was his early observation and implementation of therapeutic hypothermia, which helped to lay the foundation for modern use of such therapies [5]. One of his memoirs provides a detailed account of the effects of hypothermia, as well as temperature fluctuations, on pain and gangrene development in soldiers during wartime [12]. The cold was a well-known enemy of soldiers in battle prior to Larrey’s time, with cold-related injuries running rampant among armies across the European and Asian continents who had little knowledge of its mechanisms or how to combat it [5]. Larrey made two significant observations concerning the effect of temperature on soldiers: (1) temperature fluctuations resulted in higher rates of “congelation” and eventual gangrene, and (2) cold temperatures could serve as an analgesic and operative strategy for wounded soldiers undergoing surgical procedures or amputations [1,6,12].

The first of these observations came after Larrey noticed that soldiers reported pain symptoms related to congelation only once temperatures reached around 20 degrees, whereas temperatures 10 to 15 degrees below zero resulted in no pain or other symptoms [1,12]. Additionally, Larrey observed that soldiers who warmed themselves around fires were highly affected and rapidly developed gangrene [5,12]. These observations ultimately allowed Larrey to conclude that temperature fluctuations may be to blame for high rates of gangrene among soldiers as opposed to consistently cold temperatures [1,6]. Secondly, Larrey continued to build on the use of cold temperatures to maintain both soldiers' health and comfort. For instance, he realized that wounded soldiers had better pain management while the weather was consistently cold and that bleeding was often reduced in the presence of snow or ice [5,6]. Larrey would go on to incorporate these theories throughout his time practicing wartime medicine, improving both pain management and surgical outcomes. Though this concept is rudimentary compared to current targeted temperature management strategies, Larrey's observations represented an early recognition of the physiologic effects of temperature on pain, tissue injury, and surgical outcomes. These concepts later evolved into modern applications of therapeutic hypothermia and neuroprotective temperature management following cardiac arrest and neurologic injury [18,19].

Impact on modern surgical practice

Many of Larrey’s innovations form the basis of modern trauma and emergency care systems and practices. Modern triage protocols used in both civilian emergency departments and military combat zones reflect Larrey's prioritization strategies based on injury severity rather than social status. His triage model introduced the principle that medical intervention should prioritize injury severity and survivability rather than social rank. This philosophy remains central to modern-day civilian emergency departments, disaster response protocols, and military combat casualty care systems. 

Similarly, the concept of rapid evacuation through mobile medical units (e.g., the "ambulance volante") is directly mirrored in present-day ambulance systems and air medical transport. It established one of the earliest organized systems for the rapid evacuation and transport of injured patients from the site of injury to definitive surgical care sites. Modern emergency medical services and aeromedical services reflect many of the same operational principles. Emphasis is placed heavily on mobility and reducing treatment delays amongst these contemporary systems. 

Early amputation principles, as formulated by Larrey, emphasized rapid intervention to reduce the risk of infection, aligning with current damage-control surgery practices. His advocacy for timely surgical intervention parallels principles now seen in modern trauma and damage-control surgery. He recognized early that delayed intervention increased mortality and infection rates despite era limitations regarding anesthesia and antibiotics. 

Additionally, Larrey’s observations on hypothermia have evolved into targeted temperature management strategies used in cardiac arrest and neuroprotection. These wartime observations anticipated later developments for therapeutic hypothermia and neuroprotective care. Collectively, these parallels highlight the important and enduring influence that Larrey has on modern surgical doctrine. Larrey's contribution extended beyond battlefield improvements. They have ultimately contributed to a broader evolution of organized trauma system frameworks, emergency medicine, and modern surgical practice [4,10-16].

Legacy

After Napoleon's fall, Larrey spent the rest of his life as a writer and civilian doctor [1]. He documented many cases from his military service that led to advances in medical care for soldiers. In Napoleon’s will, he left 100,000 francs to Larrey and wrote: “he was the most courageous and virtuous man I have ever known” [3]. At the age of 72, Larrey contracted pneumonia while inspecting military hospitals in Algeria with his son. After becoming sick, he returned home to Lyons, France, where he died on July 25th, 1842 [1]. On December 15, 1992, Larrey’s remains were reburied at Les Invalides, close to Napoleon’s tomb, to honor his heroic contributions to military medicine [3].

Discussion

Baron Dominique-Jean Larrey was a pioneer in modern trauma surgery and in improving the efficiency of medical care. His inquisitive nature led to the development of efficient medical practices, subsequently improving wartime outcomes. His emphasis on rapid intervention, structured triage, and mobility altered the status quo, leading to delayed, hierarchical care. His work represents an early transition from hierarchical to needs-based care, a principle that underlies modern trauma systems.

Many of his innovations were born out of the need to manage large numbers of critically injured soldiers in resource-limited settings. However, the processes developed are now integral components of the modern healthcare system. His emphasis on prioritizing injury severity, improving evacuation efficiency, and rapidly delivering surgical intervention anticipated many operational principles that have translated to modern surgical practices. Larrey's work contributed not only to advances in early surgical techniques but also to the broader evolution of coordinated care in trauma systems. 

Larrey's contributions occurred within the context of technological and medical limitations of the 18th and 19th centuries. Lack of anesthesia and antiseptic techniques heavily influenced the outcomes. Despite these constraints, his forward-thinking approach improved survival and laid the groundwork for future advancements. Overall, his work demonstrates how innovation out of necessity can catalyze lasting transformation in modern medical practice.

## Conclusions

As modern physicians, understanding the historical foundations of trauma and emergency medicine remains important in appreciating the evolution of present-day clinical practice. Baron Dominique-Jean Larrey was a French military surgeon who revolutionized battlefield medicine during the French Revolution and Napoleonic Wars. His most important innovations included the triage system, flying ambulances, and rapid amputation techniques, all of which we still use today in both civilian and military medicine. Not only did his findings significantly improve care for soldiers during wartime, but they also laid the foundation for modern emergency and military trauma medicine. Larrey’s legacy illustrates the innovative development under extreme and resource-limited circumstances, which has transformed the structure and delivery of modern healthcare systems. 
